# Network-Based Analysis Reveals Novel Biomarkers in Peripheral Blood of Patients With Preeclampsia

**DOI:** 10.3389/fmolb.2022.757203

**Published:** 2022-06-16

**Authors:** Jing Lin, Yu Meng, Meng-Fan Song, Wei Gu

**Affiliations:** ^1^ The International Peace Maternity and Child Health Hospital, School of Medicine, Shanghai Jiao Tong University, Shanghai, China; ^2^ Shanghai Key Laboratory of Embryo Original Diseases, Shanghai, China; ^3^ Shanghai Municipal Key Clinical Specialty, Shanghai, China

**Keywords:** MS4A2, preeclampsia, WGCNA, GEO, nomogram

## Abstract

WGCNA is a potent systems biology approach that explains the connection of gene expression based on a microarray database, which facilitates the discovery of disease therapy targets or potential biomarkers. Preeclampsia is a kind of pregnancy-induced hypertension caused by complex factors. The disease’s pathophysiology, however, remains unknown. The focus of this research is to utilize WGCNA to identify susceptible modules and genes in the peripheral blood of preeclampsia patients. Obtain the whole gene expression data of GSE48424 preeclampsia patients and normal pregnant women from NCBI’s GEO database. WGCNA is used to construct a gene co-expression network by calculating correlation coefficients between modules and phenotypic traits, screening important modules, and filtering central genes. To identify hub genes, we performed functional enrichment analysis, pathway analysis, and protein-protein interaction (PPI) network construction on key genes in critical modules. Then, the genetic data file GSE149437 and clinical peripheral blood samples were used as a validation cohort to determine the diagnostic value of these key genes. Nine gene co-expression modules were constructed through WGCNA analysis. Among them, the blue module is significantly related to preeclampsia and is related to its clinical severity. Thirty genes have been discovered by using the intersection of the genes in the blue module and the DEGs genes as the hub genes. It was found that HDC, MS4A2, and SLC18A2 scored higher in the PPI network and were identified as hub genes. These three genes were also differentially expressed in peripheral blood validation samples. Based on the above three genes, we established the prediction model of peripheral blood markers of preeclampsia and drew the nomogram and calibration curve. The ROC curves were used in the training cohort GSE48424 and the validation cohort GSE149437 to verify the predictive value of the above model. Finally, it was confirmed in the collected clinical peripheral blood samples that MS4A2 was differentially expressed in the peripheral blood of early-onset and late-onset preeclampsia, which is of great significance. This study provides a new biomarker and prediction model for preeclampsia.

## Introduction

Weighted gene co-expression network analysis (WGCNA) is a popular system biology method (Langfelder and Horvath, 2008). WGCNA identifies module characteristic genes or hub genes to summarize these co-expressed gene clusters, then connects modules with phenotypes to obtain the most phenotypic trait-related modules. It may be used to discover gene clusters with strongly correlated expression modules and to investigate the gene-phenotype connection efficiently. WGCNA has a significant benefit in that it can group genes into co-expression modules and create a link between sample features and gene expression changes. Thousands of genes have been examined by WGCNA, and gene modules connected to clinical characteristics have been found. Further correlation network analysis can identify crucial and central participants in the module, thereby facilitating the discovery of therapeutic targets or candidate biomarkers. WGCNA sheds light on key genes and signaling networks that might influence disease progression (Stuart et al., 2003). It's frequently utilized in biological research, including cancer, COPD, neuropsychiatric disease, and it's a valuable tool for identifying potential biomarkers or therapeutic targets. In this study, based on the integrated microarray data set, we utilized the WGCNA approach to identify gene expression modules in the peripheral blood of preeclampsia and analyzed the key genes in these modules. This is the first time that WGCNA has been used to identify important genes in preeclampsia patients’ peripheral blood, which will facilitate the identification of biomarkers for the diagnosis and prediction of preeclampsia.

Several studies have shown preeclampsia to raise the risk of future cardiovascular disease in both mothers and fetuses. It also increases the risk of mental and neurological problems in the offspring and the developmental risks associated with preterm delivery. Pregnant women at high risk of preeclampsia should start taking aspirin at 12–16 weeks, according to the American College of Obstetricians and Gynecologists (ACOG). Preeclampsia, on the other hand, seems to have no effective treatment other than delivery. As a result, understanding the molecular mechanisms underlying the onset and progression of preeclampsia might improve practical therapy. With the advancement of technology such as big data and high-throughput sequencing, using bioinformatics to investigate the pathogenesis of preeclampsia now offers significant benefits. Our findings might lead to the development of novel peripheral blood biomarkers for the prediction and diagnosis of preeclampsia, as well as clinical intervention targets.

## Materials and Methods

### Data Acquisition and Preprocessing

**FIGURE 1:**
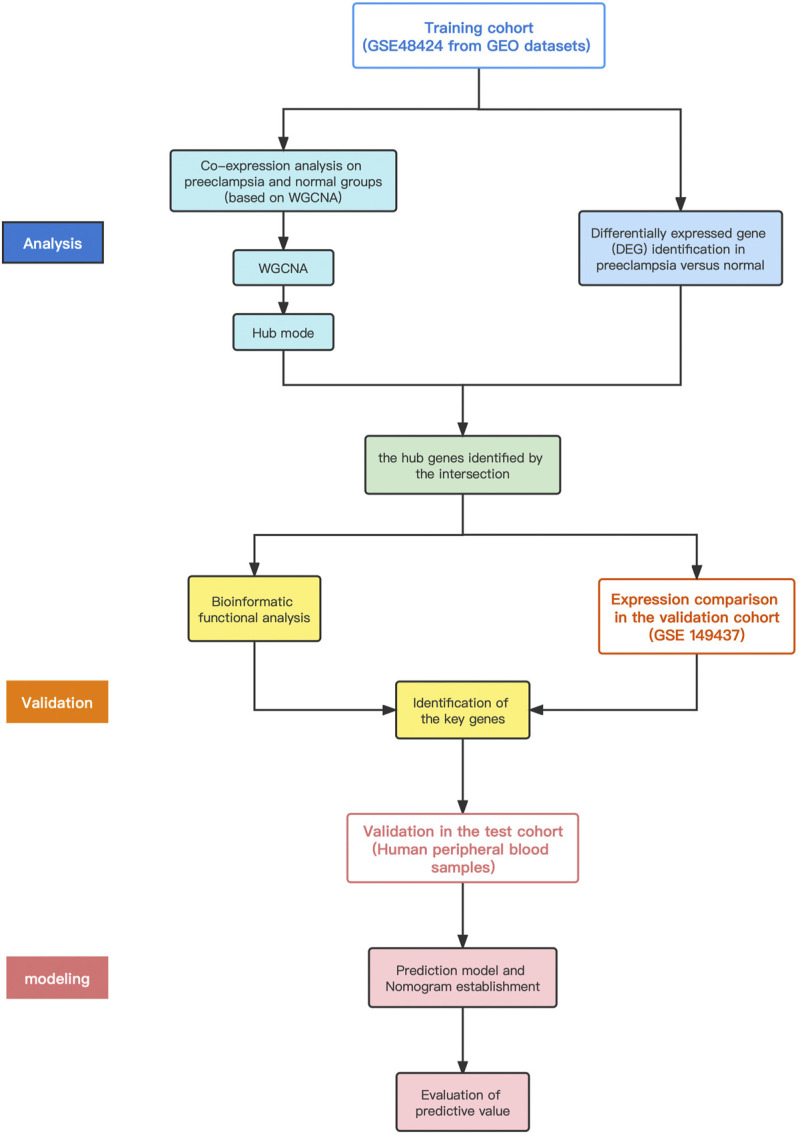
Flowchart of data preparation, analysis, and discussion in this study.

The data used in this article comes from the NCBI (Gene Expression Omnibus, http://www.NCBI.nlm.gov/GEO) GEO database. Gene Expression Integration (GEO) is the largest source of published microarray data. Look for high-throughput functional genomics experiments in preeclampsia on GEO. The selection criteria for this study are 1) Research containing peripheral blood samples of preeclampsia patients and normal pregnant women in the control group; 2) Research on gene expression profiles; 3) Publicly accessible raw data or processed data; 4) Research on *Homo sapiens*; 5) Total sample size>15. The data entry number is GSE48424, and the data comes from the work of Textoris J (Textoris et al., 2013). The platform is Agilent-014850 Whole Human Genome Microarray (GPL6480). The data set includes the peripheral blood gene expression of 36 subjects: 18 cases of PE, including 5 cases of non-severe PE, 13 cases of severe PE, and 18 cases with normal pregnancy. The data analysis program is shown in Figure 1. We first download the normalized data, obtain the expression matrix, and filter the data to remove probes without corresponding annotation information. After data preprocessing, we retained 30,922 genes for further analysis.

### Data and Statistical Analysis

The GSE48424 data set comes from the GEO website. The limma software package is used in R studio for quality control, preprocessing, and statistical analysis. The data is normalized by the robust multi-array averaging (RMA) method, and WGCNA analysis is performed on the entire GSE48424 data set.

### Construction of Weighted Gene Co-expression Network and Identification of Significant Modules

The data were processed using R-Studio 3.4.0 software. To ensure that the results of network construction were reliable, abnormal samples were removed. The WGCNA package was used to construct the co-expression network (8). First, the samples were clustered in order to assess the presence of any significant outliers. Second, the co-expression network is constructed using the automatic network construction function, which uses the R function to pick up the soft threshold to calculate the soft threshold setting power *β*. The similarity of co-expressions is proposed to calculate the adjacency. To produce a tree diagram, hierarchical clustering is performed to each block, and the modules are designated as branches. Automatic module merging was performed for modules with highly correlated feature genes (max Block Size = 6000, T0M Type = ‘unsigned’, min module Size = 25, merge Cut Height = 0.25). As a result, genes with comparable expression patterns were grouped together into a single module, and each such module was given a color. For modules associated with clinical attributes, module affiliation (MM) and gene significance (GS) were calculated. The gene information in the modules is used for further analysis. For further investigation, the appropriate module gene information was extracted. Finally, the characteristic gene network was visualized.

For each module, eigengene represents the main gene expression pattern in its constituent gene members. Gene module membership measures how close the expression pattern of a gene is to its eigengene. It is expected that genes with high module membership will have more important functions in the module and are more likely to be related to clinical traits. In turn, if the genes in a module show a high correlation between module membership and gene importance (association with traits), this suggests that the connection between the gene module and clinical characteristics is extremely reliable. To this end, we mapped the membership and gene significance of the gene modules of the modules related to clinical traits.

### Differentially Expressed Genes Screening and Venn Diagram

We screened differentially expressed genes (DEGs) in preeclampsia patients and control groups using the “limma” R software package. To select genes for further consideration in the network construction, the significance analysis of microarrays (SAM) with false discovery rate (FDR) < 0.05 and |log2 fold change (FC)| ≥ 1 was applied. Use the Venn Diagram package in R to make the results of GEO differential expression analysis and the WGCNA blue module into a Venn diagram. The intersection of WGCNA phenotype-related modular genes and DEGs is displayed and visualized in the Venn diagram (http://bioinfogp.cnb.csic.es/tools/venny/index.html), representing disease-related genes and differentially expressed genes. These 30 overlap genes were considered to be hub genes and the expression of these genes is shown in Table 1.

**TABLE 1:** The gene expression levels of 30 overlap hub genes.

Gene Symbol	*p* value	logFC	Gene title
PLD1	0.000002	−1.53645	Phospholipase D1
MME	0.000010	−1.29260	Membrane metallo-endopeptidase
TMEM165	0.000025	−1.31920	Transmembrane protein 165
ZDHHC21	0.000038	−1.05659	Zinc finger DHHC-type containing 21
CCNG2	0.000072	−1.14684	Cyclin G2
F2RL1	0.000079	−1.44107	F2R like trypsin receptor 1
CXCL6	0.000162	−1.19486	C-X-C motif chemokine ligand 6
CPEB2	0.000193	−1.62313	Cytoplasmic polyadenylation element binding protein 2
MAP4K3	0.000258	−1.05478	Mitogen-activated protein kinase kinase 3
PPM1H	0.000282	−1.18759	Protein phosphatase, Mg2+/Mn2+ dependent 1H
HDC	0.000302	−1.70069	Histidine decarboxylase
PHIP	0.000418	−1.62474	Pleckstrin homology domain interacting protein
SLC18A2	0.000544	−1.14731	Solute carrier family 18 member A2
SCML1	0.000633	−1.21133	Sex comb on midleg-like 1 (*Drosophila*)
COL4A3	0.000767	−1.15475	Collagen type IV alpha 3 chain
SPDYE1	0.000851	−1.19125	Speedy/RINGO cell cycle regulator family member E1
SENP7	0.000944	−1.14301	SUMO1/sentrin specific peptidase 7
ZNF711	0.001070	−1.25791	Zinc finger protein 711
LOC643441	0.001240	−1.16769	Uncharacterized LOC643441
MGAM	0.001590	−1.01793	Maltase-glucoamylase
SLC37A3	0.001690	−1.05883	Solute carrier family 37 member 3
CUX2	0.001770	−1.19767	Cut like homeobox 2
MS4A2	0.002080	−1.23060	Membrane spanning 4-domains A2
HOOK1	0.002580	−1.01997	Hook microtubule tethering protein 1
FAM126B	0.002620	−1.20200	Family with sequence similarity 126 member B
SLC4A10	0.003680	−1.29071	Solute carrier family 4 member 10
CASD1	0.004040	−1.01822	CAS1 domain containing 1
PLA2G7	0.004290	−1.06796	Phospholipase A2 group VII
NCOA2	0.004520	−1.12527	Nuclear receptor coactivator 2
SNX16	0.008180	−1.03120	Sorting nexin 16

### Protein-Protein Interaction (PPI) Network Construction and Identification of Hub Genes

The previously acquired genes from each module are mapped to the search platform STRING database (STRING, V11.0; https://string-db.org/) to comprehensively identify the hub genes of each module and the distinctive genes of the modules. It is essential in the protein-protein network (PPI). The CytoHubba plugin, which is based on the Cytoscape software (http://www.cytoscape.org/, version 3.7.1; Institute for Systems Biology, Seattle, WA, United States), was then used to construct and visualize the protein interactions of each module, with the highest degree of connection being identified as the hub gene. MCC is considered to be the most accurate of all available methods in CytoHubba (Baralić et al., 2020). Considering that it can generate accurate predictions of essential proteins, MCC is used.

### Functional Enrichment Analysis and KEGG Analysis of Core Genes

The 30 overlap hub genes were uploaded to DAVID6.8 (http://david-d.ncifcrf.gov/) for functional enrichment analysis. Gene ontology (GO) analysisis is used to determine differentiating biological characteristics. To determine functional attributes, the Kyoto Encyclopedia of Genes and Genomes (KEGG) pathway enrichment analysis was used. The significance setting at *p* < 0.05 is considered to be significant enrichment. GO function annotation includes three aspects: biological process (BP), molecular function (MF), and cell component (CC). In addition, the Kyoto Encyclopedia of Genes and Genomes (KEGG) enrichment pathway analysis was carried out on kobas3.0 (http://kobas.cbi.pku.edu.cn/) (Bu et al., 2021). All results are expressed as adjusted *p* value < 0.05 and visualized by R software.

### Validation of the Hub Genes Expression and Prediction Value

GSE149437 contains 66 cases of preeclampsia and 376 cases of non-preeclampsia whole blood samples of different gestational weeks. The current guidelines recommend taking aspirin from 12 weeks of pregnancy to prevent preeclampsia. In order to verify the difference in peripheral blood gene expression of preeclampsia in the first trimester, we selected the data of all pregnant women whose gestational age is 10^+0^–12^+6^ weeks in GSE149437 to verify the expression difference of hub genes. Box plots were constructed using “ggplot2” package in R software (version 3.6.3) to analyze the hub gene expression in the blood samples between preeclampsia and non-preeclampsia women in gene expression profile. Data are expressed as standard deviation. Unpaired independent *t*-test was used for statistical analysis, and *p* < 0.05 was set as the critical value to consider the statistical significance.

### Establishment and Validation of Prediction Models and Nomogram

We used logistic regression analysis to establish the prediction model. Hub genes that were differentially expressed in the training cohort and validation cohort would be included in the multivariate model. The nomogram was also developed based on the regression coefficients of the relevant genes in the training cohort. The values for model covariates were mapped to points in the range of 0–100. The total number of points obtained by the predictive model corresponded to the risk of preeclampsia. The performance of the nomogram was evaluated by the calibration curve in the training cohort. In both the training cohort and validation cohort, the prediction ability of the model is evaluated by the area under the ROC curve (AUC). ROC curves were generated using SPSS 22.0 (IBM, Corp.). The genes were regarded to have potential clinical significance if the area under the ROC curve (AUC) was greater than 0.6.

### Acquisition of Human Blood Samples and Hub Genes Validation

Human peripheral blood samples were obtained from patients who underwent prenatal examination at the International Peace Maternal and Child Health Hospital during the first trimester of pregnancy. Fasting blood samples were collected from the pregnant woman after one night of random fasting (10–12 weeks of pregnancy). All specimens were obtained after the patients provided informed consent. The samples were promptly frozen in liquid nitrogen for further experiments. The Ethics committee of International Peace Maternal and Child Health Hospital approved the use of these samples for total RNA isolation and real-time polymerase chain reaction (RT-PCR) analysis. Information on pregnancy outcomes was obtained after delivery and grouped. The test cohort included 30 samples (10 control groups, 11 late-onset, and 9 early-onset preeclampsia groups). The clinical information of the patients is shown in Table 2.

**TABLE 2:** Comparison of demographic and characteristics in the test cohort.

Characteristic	Control (*n* = 10)	Late-onset Preeclampsia (*n* = 11)	Early-onset Preeclampsia (*n* = 9)
Gravidity, n (%)	—	—	—
1	6 (60.0%)	7 (63.6%)	3 (33.3%)
2	2 (20.0%)	2 (18.2%)	4 (44.4%)
3	1 (10.0%)	1 (9.1%)	2 (22.2%)
4	1 (10.0%)	1 (9.1%)	0 (0%)
Parity, n (%)	—	—	—
0	9 (90.0%)	10 (90.9%)	6 (66.7%)
1	1 (10.0%)	1 (9.1%)	1 (11.1%)
Education, n (%)	—	—	—
Bachelor’s degree	6 (60.0%)	7 (63.6%)	3 (33.3%)
Master’s degree	1 (10.0%)	3 (27.33%)	5 (55.6%)
Senior middle school	3 (30.0%)	1 (9.1%)	1 (11.1%)
Age (y)	30.6 ± 3.9	31.0 ± 3.9	33.9 ± 4.8
BMI (kg/m^2^)	22.3 ± 2.6	22.5 ± 3.8	22.4 ± 3.3

Measurement data is expressed by mean ± SD., Count data is expressed by number (%).

### Quantitative RT-PCR Analysis

After obtaining the hub gene through the co-expression network, we verified it by RT-PCR. We collected 10 peripheral blood sample sure, including 5 normal controls and 5 pre-eclampsia blood samples. Follow the manufacturer’s recommendations to synthesize cDNA using TransScript One-Step gDNA Removal and cDNA Synthesis SuperMix (Trans, Beijing). SYBR Green Mix (QIAGEN, Germany) and specific primer sets were used to perform RT-PCR on the obtained cDNA. The PCR product was verified by melting curve analysis. The delta-delta Ct method was used to calculate the relative quantitative expression, and each gene was normalized to GAPDH. Three biological replicates and three technical replicates were used for analysis. The primers and primer sequences of each gene are shown in supplementary information 1.

### Statistical Analysis

The statistical significance was determined using the *t*-test and One Way ANOVA tests of R software. The statistical analyses were performed in R v.3.5.1, and *p* < 0.05 is considered a statistically significant difference.

## Results

### Soft Threshold Calculation

**FIGURE 2:**
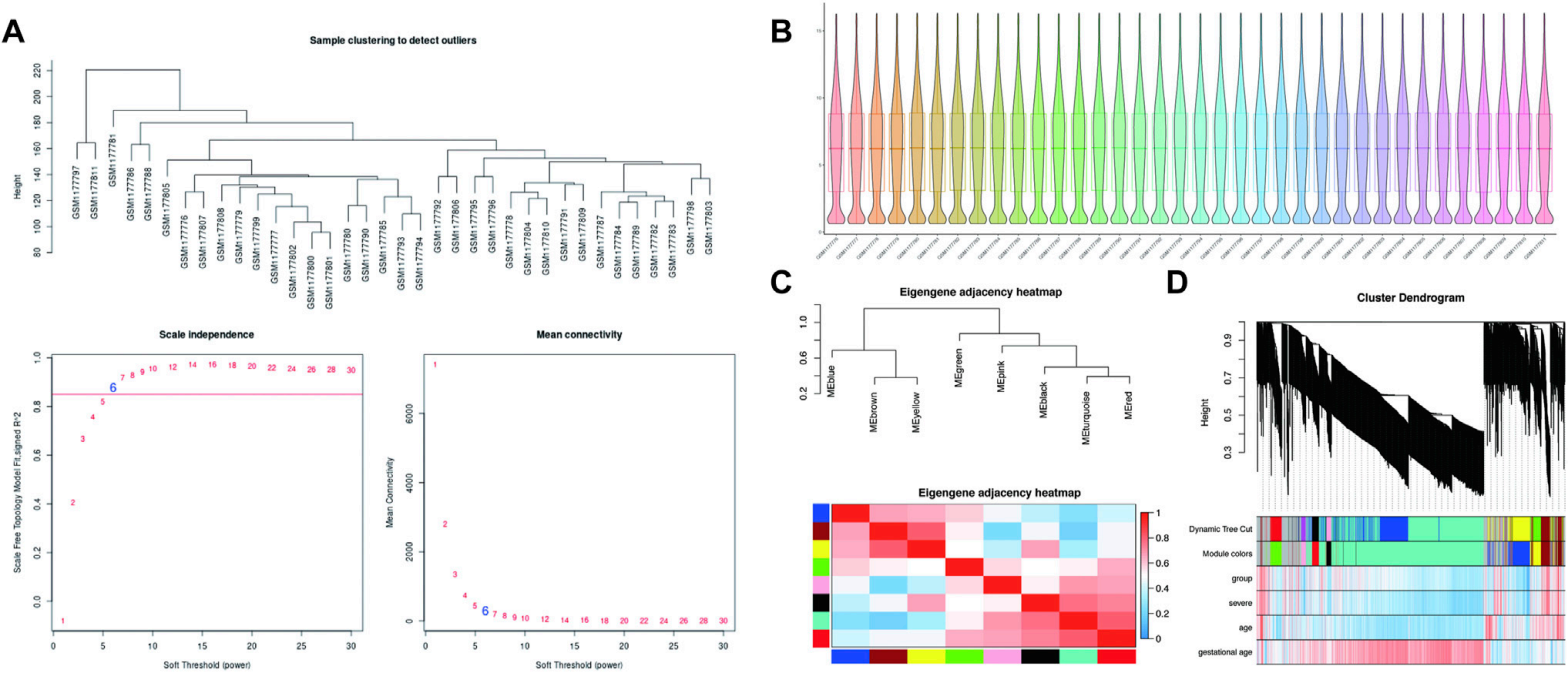
WGCNA processing for preeclampsia data set GSE48424. **(A)** Determination of soft-thresholding power. **(B)** Box plots illustrating data normalization. **(C)** Module eigengene adjacency heatmap. Module-eigengenes (ME) are defined as the first principal component of a coexpression module matrix. The heatmap shows the relatedness of co-expression gene modules identified by WGCNA (red, positive correlation; blue, negative correlation). Color scale indicates the range of correlation coefficients. The correlation coefficient is between 1 and +1, where 1 indicates the strongest possible correlation and 0 indicates the weakest possible correlation. **(D)** WGCNA correlation network results in preeclampsia. Clustering dendrogram of species, with dissimilarity determined by topological overlaps, along with assigned module colors. Weighted gene coexpression network analysis (WGCNA) can be used to group genes into 9 different gene modules based on their co-expression patterns.

The soft-thresholding power is calculated to make the network more in line with the characteristics of a scale-free network. The optimal value of the soft-thresholding power needs to make the adjacency function better satisfy the scale-free condition, and generally, R^2^ > 0.8 satisfies the scale-free condition (Ullah, 2019). In this data set, when the soft-thresholding power is 6, the R^2^ of scale-free network spectrum structure reaches 0.85, which ensures that the network is close to the scale-free network (Figure 2A). It is also the minimum threshold for smoothing the curve, and it keeps the network’s average connection degree from being too low, allowing the network to store adequate information.

### Co-Expression Networks

In theory, the genes are classified according to the expression pattern by computing the correlation coefficient between them, and the patterned genes are grouped into a module. Through WGCNA analysis, 9 co-expression modules were constructed, ranging in size from 36 to 1060 genes (each module was assigned a color for reference) (Table 3). The clustering dendrogram of genes is shown in Figures 2C,D. The clustering dendrogram is based on the topological overlap. Genes with similarities are clustered in one module color. Moreover, these modules were independent of other modules.

**TABLE 3:** Module and the number of genes in each module.

Turquoise (1060)	Grey (345)	Blue (158)	Brown (135)	Yellow (81)
green (79)	Red (62)	Black (38)	Pink (36)	—

### Modular-Trait Correlation Analysis and Hub Gene Identification in Preeclampsia

**FIGURE 3:**
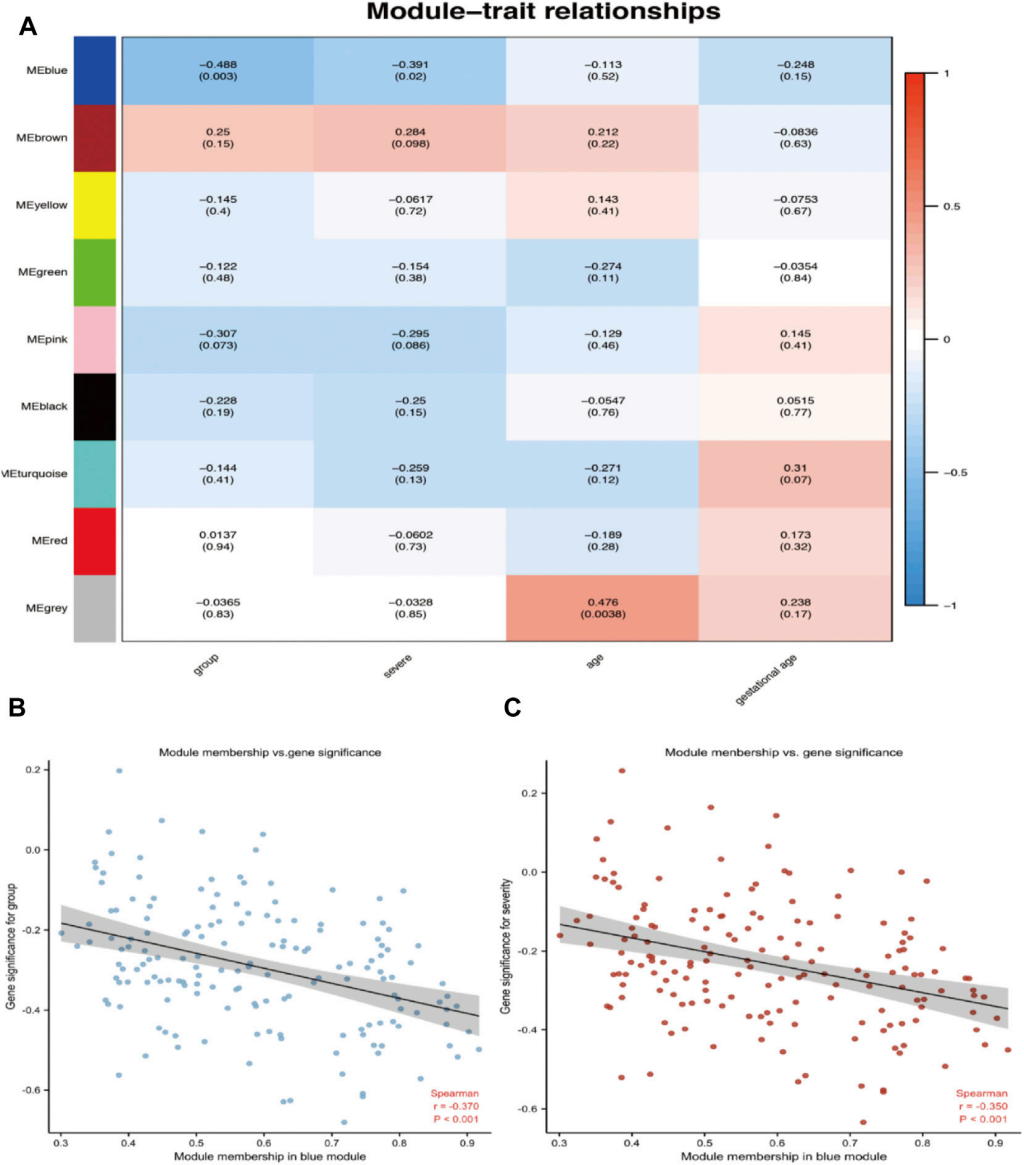
Target module screening and analysis. **(A)** Detection of gene modules associated with clinical traits. Correlation matrix between each module and preeclampsia. Each module is assigned a color. Then, each module is tested for correlation with the onset and severity of preeclampsia. Cell colors encode correlation coefficients (red, positive correlation; blue, negative correlation). The color scale indicates the range of correlation coefficients. The number in parentheses indicates the corresponding *p*-value. **(B,C)** The correlation between the module membership (MM) and gene significance (GS) of the disease group **(B)** and the severity **(C)** of all genes in the blue module. Spearman r represents the absolute correlation coefficient between GS and MM.

An advantage of co-expression network analysis is the capacity to integrate external information. This study focused on the relationships between gene modules and preeclampsia. The significance of the module could be determined as the average absolute gene significance index. After the procedures mentioned above, the color intensity was identified to be proportional to the disease status and severity. Figure 3 shows a summary of the significance of all genes in each module related to preeclampsia. It also clearly shows that the blue module is most significantly related to preeclampsia and is related to disease severity. Therefore, these genes can be regarded as hub genes.

### Gene Ontology Functional Analysis

**FIGURE 4:**
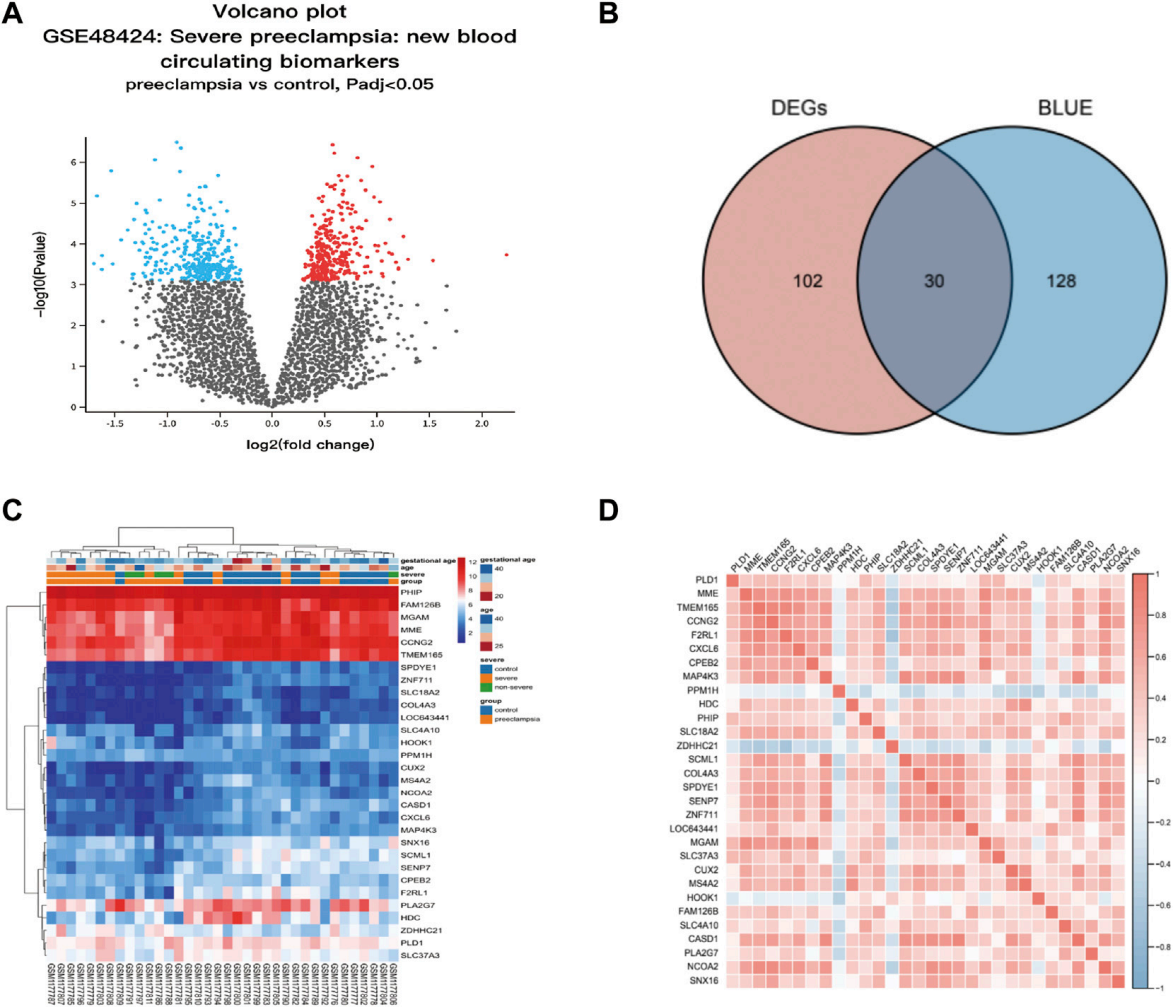
Hub genes identification from the overlap of DEGs and WGCNA. **(A)** The volcano plot for differentially expressed genes (DEGs) between normal and preeclampsia samples in GSE48424. **(B)** The overlap of differentially expressed genes (DEGs) and hub genes was shown as a Venn diagram. Identification of common genes between differentially expressed genes (DEGs) and the blue module overlapping them. **(C)** Heatmap of 30 hub genes between patients with preeclampsia and the control individuals; Each row represents a sample number, and each column represents a single gene. The gradual color change from red to blue represents the changing process from upregulation to downregulation. **(D)** Susceptibility genes to preeclampsia were identified.

**FIGURE 5:**
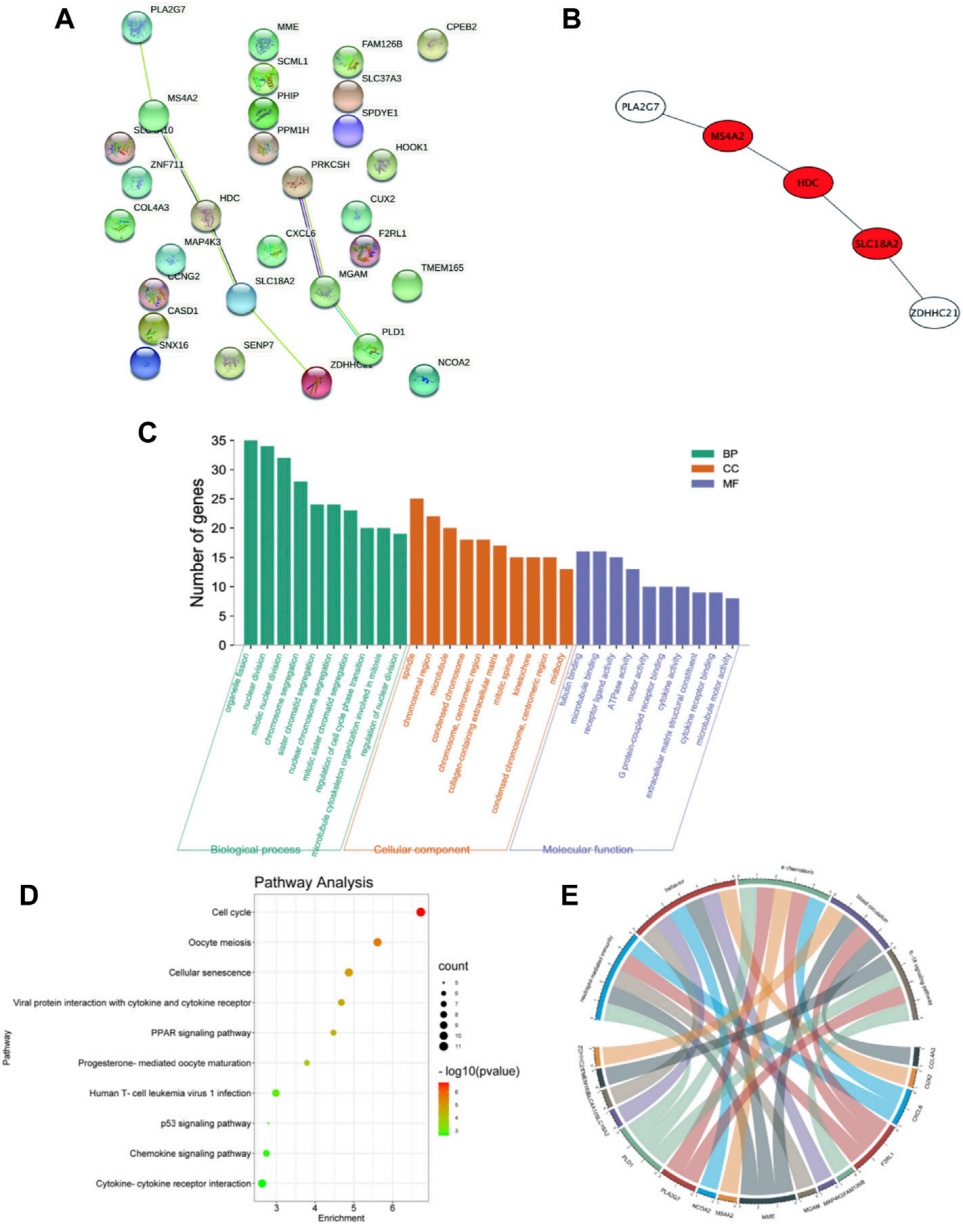
PPI network and functional enrichment analysis of hub genes. **(A)** PPI network of 30 hub genes generated by the Cytoscape software. STRING was applied to create PPI network to define protein interactions. **(B)** The top 3 hub genes with the most correlations identified using CytoHubba, and the core gene scores were calculated using the Maximal Clique Centrality (MCC) method. **(C)** GO enrichment analysis results for hub genes. The functional enrichment analysis was performed using the Database for Annotation, Visualization and Integrated Discovery (DAVID). GO terms enrichment for hub genes are shown in biological processes, molecular function, and cellular components. Y-axis shows the number of genes, and the x-axis shows the terms of the GO pathway. **(D)** The Kyoto Encyclopedia of Genes and Genomes (KEGG) pathways enrichment: The sizes of the circle dots indicate the numbers of enriched genes, and the color of circle color indicates the *p*-value. The x-axis represents the number of genes in enrichments. **(E)** The GO-enriched chord diagram shows the genes involved in the GO term.

Thirty candidate genes were identified from the intersection of a venn diagram between two sets of DEGs and the WGCNA blue module (Figure 4). GO and KEGG pathway enrichment analyses were performed using the “clusterProfiler” package in R software to explore the biological features and significance of the 30 hub genes (Figure 5). In biological processes (BP), clusters are significantly related to organelle fission, nuclear division, and mitotic nuclear division. In molecular function (MF) analysis, our results indicate that hub genes are significantly related to the spindle, chromosomal region, and microtubule in molecular function (MF) analysis. Cell component (CC) enrichment analysis was found to focus on tubulin-binding, microtubule-binding, receptor-ligand activity. In KEGG pathway analysis, cell cycle, oocyte meiosis, and cellular senescence signaling pathway were found to be the significant pathways in 30 hub genes. The findings revealed that these genes were significantly enriched in cell cycle-related pathways, implying that they may play a role in cell division.

### Extract Hub Genes From DEGs and the Hub Module in WGCNA

For further study, the protein-protein interaction (PPI) network was constructed among 30 candidate genes (string, https://string-db.org/), and Cytoscape software was applied to visualize the PPI network. The potential key genes were identified based on Maximal Clique Centrality (MCC) through CytoHubba plug-in (Figure 5). The top 3 Hubba nodes were collected for subsequent analysis. Among all 30 genes, we found SLC18A2, HDC, and MS4A2 were identified as the hub gene by the CytoHubba plug-in.

### Validation of Hub Gene in GSE149437 and Establishment of a Prediction Model

**FIGURE 6:**
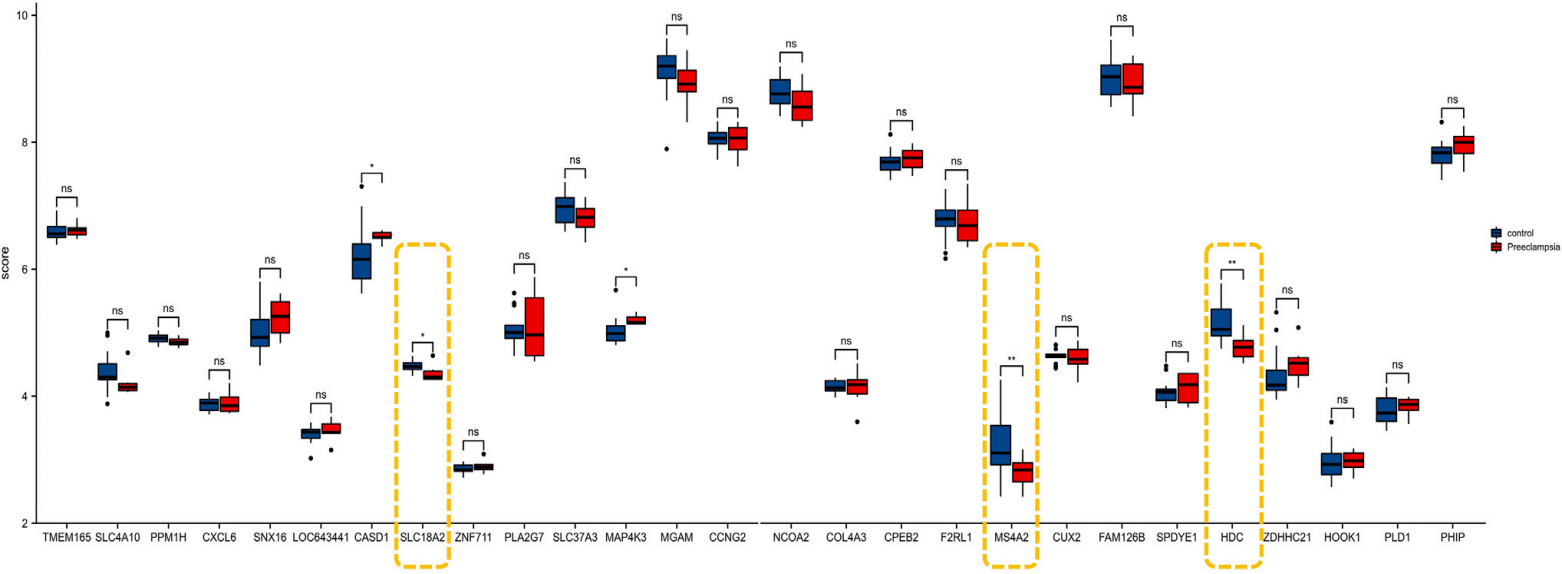
The expression of 30 genes in the validation cohort (GSE149437).

**FIGURE 7:**
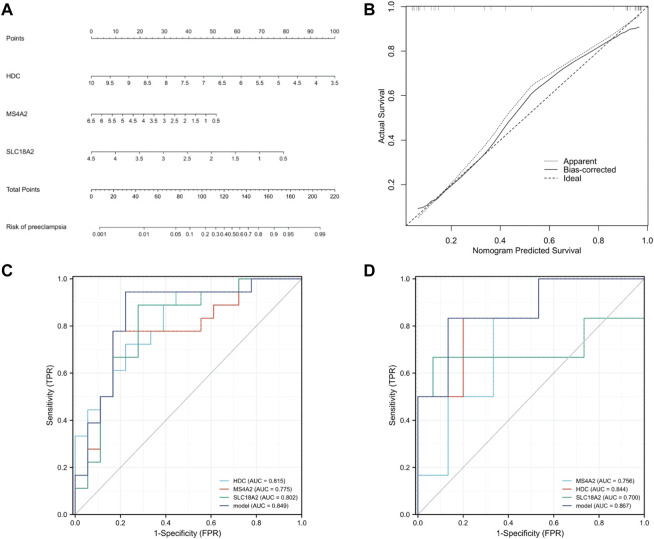
Establishment of nomogram and model validation. **(A)** A nomogram was used to estimate the risk of preeclampsia in the training cohort. To calculate the probability of preeclampsia, draw a line perpendicular to the corresponding axis of each risk factor until it reaches the marked point of the score line at the top. The probability of preeclampsia can be indicated by summing the scores of all risk factors and then positioning it on the corresponding bottom line. **(B)** Calibration curve of training queue. The probability of the nomogram predicting preeclampsia is plotted on the x-axis; The actual probability of preeclampsia is plotted on the y-axis. The diagonal dotted line represents the perfect prediction of the ideal model. Solid lines represent the performance of nomographs. The closer this line is to the diagonal dotted line, the better the prediction effect is. **(C)** The ROC curves of HDC, MS4A2, SLC18A2, and model in the training cohort (GSE48424). **(D)** The ROC curves of HDC, MS4A2, SLC18A2, and model in the validation cohort (GSE149437).

To further prove the significance of the key genes in the module of interest, we detected the expression of the 30 hub gene by using the whole blood samples of the early pregnancy in the GSE149437 data set. GSE149437 contains 6 cases of preeclampsia and 15 cases of non-preeclampsia whole blood samples in early pregnancy (10^+0^–12^+6^ weeks). Independent studies between the two samples showed that there were three genes with reduced expression and statistical differences in preeclampsia: SLC18A2, HDC, and MS4A2 (Figure 6). It is consistent with the above MCC results, indicating that these three genes have an important correlation with preeclampsia. Therefore, we established a prediction model of preeclampsia in the training cohort based on the expression of the above three genes. The final model we obtained was prediction model = 6.9884–0.8587*HDC +0.1018*MS4A2 -0.9388*SLC18A2. At the same time, we establish a nomogram to visualize the model and use the calibration curve to verify the accuracy of the model. The nomogram was shown in Figure 7A. The calibration curve of the training cohort was shown in Figure 7B (Mean absolute error = 0.029). The calibration curve of the nomogram for the prediction of preeclampsia risk was proven to be in good agreement. Hosmer-Lemeshow test for the evaluation of the model showed that the Chi-square value was 10.935 (*p* = 0.205 > 0.05) of the predictive model. Then we compared the value of the model prediction and three genes prediction alone. The ROC curves in Figures 7C,D showed that the value of combined three-gene prediction is higher than that of single gene prediction (AUC = 0.849 in the training cohort and AUC = 0.867 in the validation cohort). Therefore, the model we established has a good predictive value for preeclampsia.

### Validation in the Test Cohort

**FIGURE 8:**
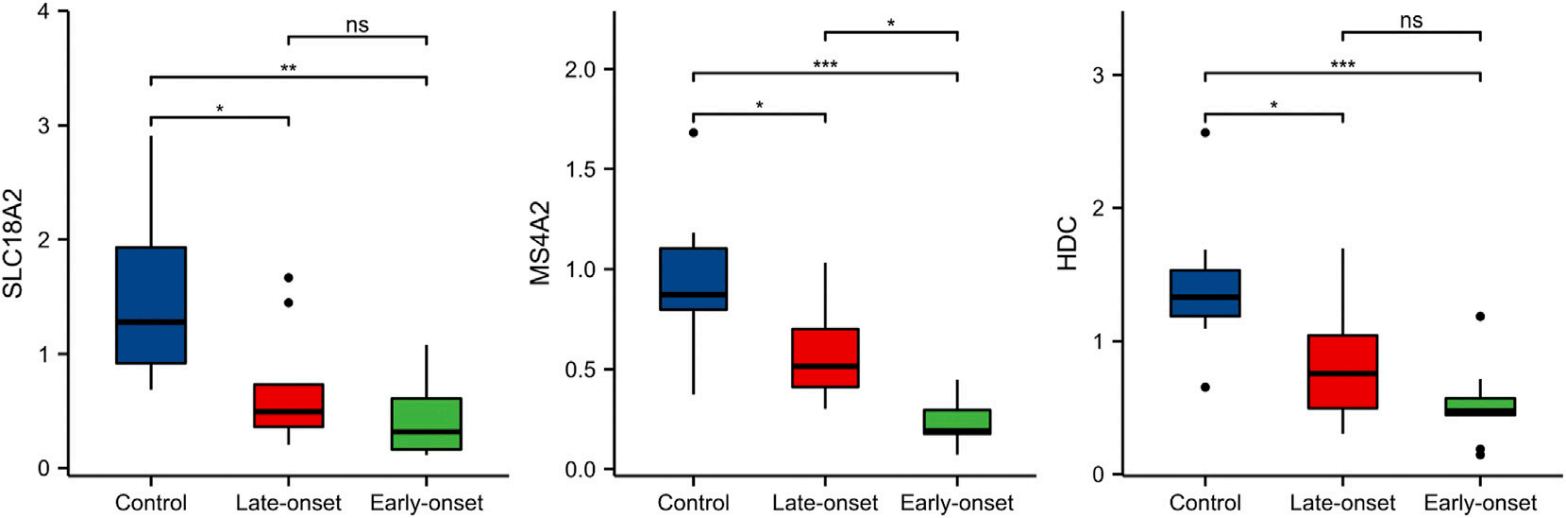
The expressions of HDC, MS4A2, and SLC18A2 in the test cohort.

To confirm the clinical value of the three genes as biomarkers in peripheral blood of patients with preeclampsia, we also established a test cohort and included 30 subjects (10 normal pregnant women, 11 late-onset preeclampsia, and 9 early-onset preeclampsia). The levels of the three genes in peripheral blood samples were detected by PCR. The results were shown in Figure 8. All the three gene expressions decreased significantly in the preeclampsia group, but only the level of MS4A2 was related to the severity of the disease.

## Discussion

Preeclampsia (PE) is a major cause of maternal and fetal morbidity and mortality worldwide. However, the fundamental molecular mechanism of preeclampsia remains unknown (Kang et al., 2021). The process underlying placental malfunction is extremely complex. Traditional methods of processing high-throughput data to detect differential molecular biomarkers based solely on group level are inefficient. In this study, we conducted multi-omics WGCNA. The co-expression network is built by combining similarly expressed probes into related network modules with potential common functionalities. Unlike other network analysis methods that focus on non-weighted networks, WGCNA is more comprehensive and qualified to construct meaningful networks whether weighted or non-weighted (Langfelder and Horvath, 2008). As a result, the analysis results are reliable and meaningful. WGCNA divides the function-related genes into a module to classify the related biological modules for further analysis. This study attempts to identify a set of genes that can provide insight into the genetic basis of preeclampsia. Using gene expression data from the peripheral blood of patients with preeclampsia, we created a gene co-expression network and correlated them with clinical variables. Due to the multiple functions of genes, it is challenging to determine the exact mechanism of preeclampsia. Therefore, WGCNA is based on biological and medical backgrounds to endow these genes with clinical significance and cluster characteristic genes according to specific pathological processes (Huang et al., 2020). Using WGCNA we found a blue module, which is significantly related to the onset and severity of preeclampsia. These characteristic genes can predict the onset of preeclampsia early, distinguish different degrees of severity, and play a vital role in the pathogenesis of preeclampsia. Then 30 key genes were screened out by taking the intersection between the differential gene and the blue module. To further explore the biological characteristics and significance of these 30 central genes, GO and KEGG analysis showed that these genes were mainly enriched in cell cycle-related pathways during the development of preeclampsia, suggesting that these genes may play a role by affecting cell division. Among these genes, we screened the key gene MS4A2 most related to preeclampsia through the database and clinical data validation.

Preeclampsia (PE) is one of the complex obstetric syndromes. Hypertension and proteinuria are critical clinical symptoms of preeclampsia. The pathogenesis is currently unclear (Fisher, 2015). It can cause intrauterine growth restriction, premature delivery, premature rupture of membranes, late spontaneous abortion, and placental abruption. Without intervention, this situation may develop into eclampsia and even endanger the lives of mothers and children. Only symptomatic treatment conditions are available. Termination of pregnancy is still the only therapeutic intervention. Their common features are related to defects in the deep placenta and relatively shallow depth of trophoblast invasion into the uterus compared to normal pregnancy. This is often accompanied by abnormal physiological transformations of spiral arteries. Immune system disorders may lead to reproductive failure or pregnancy complications (Harris et al., 2019). In this regard, recent research have improved our understanding of the mechanism that regulates immune tolerance during pregnancy. Our understanding of the function of the immune system in the development of preeclampsia has changed throughout time.(Rambaldi et al., 2019). From a model based on cell-mediated immune changes, research has turned to a vision centered on humoral immune changes and systemic involvement of the inflammatory system.

**FIGURE 9:**
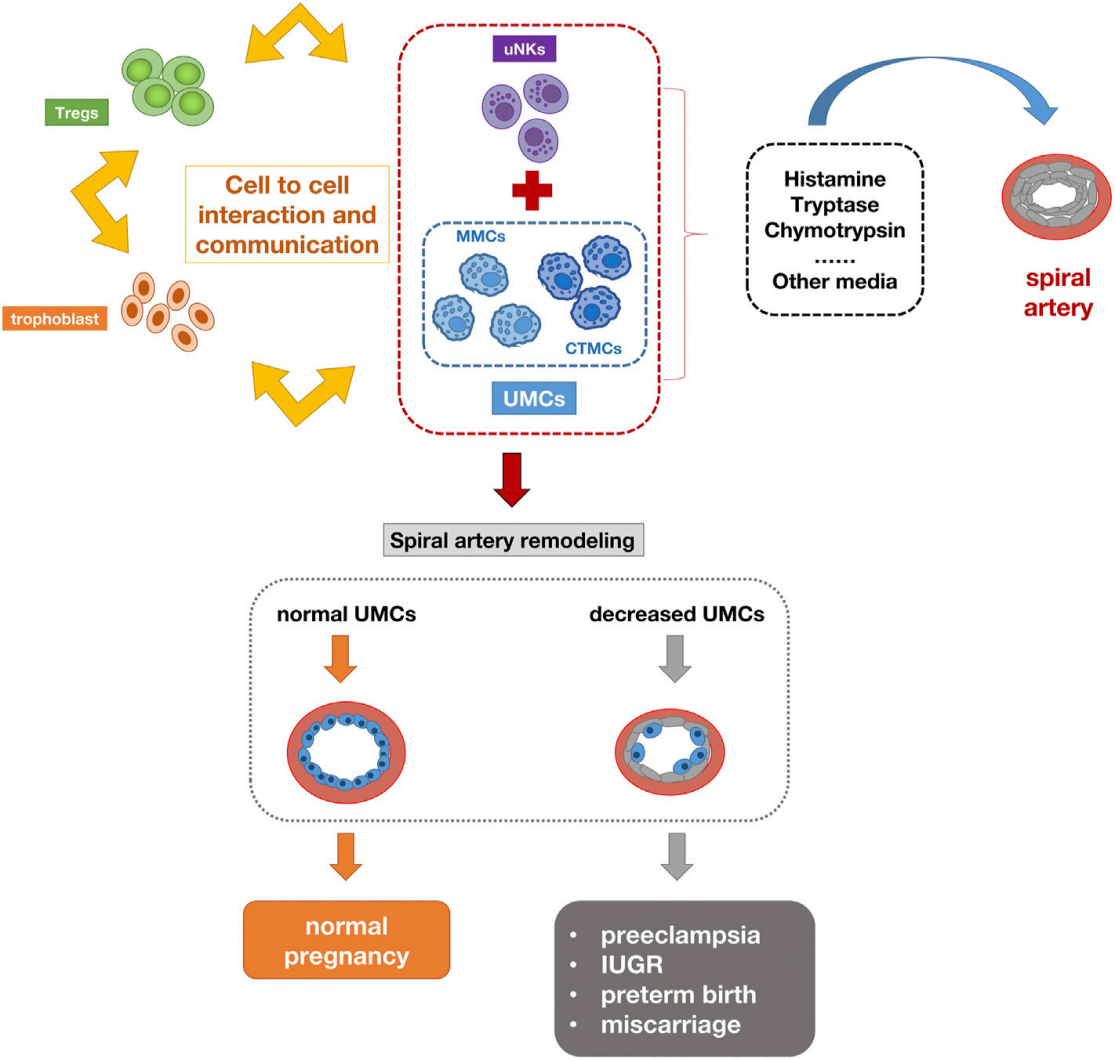
Schematic diagram of the pathogenesis of preeclampsia caused by mast cell reduction. The interaction between uMCs, uNKs, trophoblasts and regulatory T cells promotes pregnancy synergistically. The activation of uMCs and uNKs can release many mediators, including histamine, tryptase, chymotrypsin. These mediators are directly or indirectly involved in the process of uterine spiral artery remodeling. The deficiency of UMCs can lead to insufficient remodeling of the uterine spiral artery and have a negative impact on pregnancy.

During pregnancy, the maternal organism is affected by tremendous endocrine and immune changes to adapt to the implanted and developing embryonic fetus. Researchers believe that the immunological changes in the early placental microenvironment may be involved in the induction of preeclampsia. Numerous immune cells include natural killer cells, macrophages, lymphocytes, dendritic cells, and mast cells (MCs). Their imbalance can lead to pregnancy complications (Bulmer et al., 2009). Placental mast cells play multiple roles in regulating tissue remodeling, angiogenesis, trophoblast invasion, and spiral artery regulation during pregnancy. A large number of MCs were detected in the uterus during pregnancy (Menzies et al., 2012). The MC density in pregnant women was significantly higher than that in non-pregnant women (Garfield et al., 2006). Studies have shown that MCs play an important role in placental implantation and uterine vascular remodeling (Dudeck et al., 2019). Activated mast cells have been shown to contribute to arteriogenesis and angiogenesis (Bot et al., 2020). The interaction between UMCs (uterine mast cells), UNKs (uterine natural killers), trophoblasts and regulatory T cells promotes pregnancy synergistically. The chymases secreted by UMCs and UNKs are essential for supporting vascular changes required for pregnancy (Burke et al., 2008). The combined deficiency of UMCs/UNKs can have a negative impact on pregnancy, resulting in reduced placental size and shallow implantation. UMCs represent a unique group composed of MMCs (mucosal type MCs) and CTMC (connective tissue-type mast cell), as well as the third intermediate MC group (Woidacki et al., 2013a). One of the most important milestones in pregnancy is the remodeling of the spiral artery (SA), which is a key adaptation to pregnancy. Adequate SA remodeling is important for placental and fetal development, while impaired SA remodeling is associated with preeclampsia, IUGR, preterm birth, and miscarriage. For a long time, it has been considered that UNKs in innate immune cells are closely related to remodeling. However, some studies have found that their deletion or depletion does not have a far-reaching impact on pregnancy (Anne Croy et al., 2006). Recent studies have shown that UMCs play an unexpected key role in remodeling and fetal survival. A balance needs to be maintained between UNKs and UMCs to ensure SA reconfiguration (Meyer et al., 2017b). MCs activation leads to the release of a large number of pre-formed or newly synthesized mediators, including histamine, tryptase, chymase and many other mediators. These are directly or indirectly involved in processes such as placental implantation, angiogenesis, defense against pathogens and uterine remodeling, which are important for pregnancy success. For example, MCP-5 (mast cell protease-5) positively affects SA remodeling by activating vascular smooth muscle cell (VSMC) apoptosis and extravillous trophoblast (EVT) migration. Some studies also indicate that compared with the physiological placenta, the mast cell area in the placenta of preeclampsia is significantly reduced, and the degree of vascularization of the placenta is relatively low, suggesting that mast cells lose their original angiogenesis potential (Szewczyk et al., 2012). Studies on mice deficient in mast cells have demonstrated that the size of the implantation site is reduced compared to control mice with sufficient mast cells. The lack of mast cells in pregnant mice leads to reduced spiral artery remodeling, fetal growth restriction, and reduced placenta size (Woidacki et al., 2013b). *In vitro* studies have shown that the conditioned medium of mast cells increases trophoblast migration (Meyer et al., 2017a), indicating the role of mast cells in the invasion of trophoblasts in the placental bed, and the reduction of cell migration in preeclampsia leads to the occurrence of shallow placental implantation. The decrease of mast cells can indicate the severity of the disease (Broekhuizen et al., 2021). According to above reports, the number of mast cells in the placental villi of preeclampsia is reduced. These findings are related to the low degree of placental vascularization in patients with preeclampsia, suggesting the lack of placental mast cell function. MS4A2 (Membrane spanning 4-domains A2) is a marker of mast cells. This gene encodes a subunit of the IgE receptor protein and is a component of the high-affinity IgE receptor. Co-immunoprecipitation experiments have shown that it binds to FcɛRIα and FcRγ and forms a tetrameric receptor complex (αβγ_2_). The presence of MS4A2 in tetrameric FcRI has been demonstrated to provide higher structural integrity and stability to the cell surface FcRI receptor complex, in addition to its signaling amplification function (Donnadieu et al., 2000). It is expressed at high density on mast cells and basophils and participates in activating IgE-dependent mast cells and basophils. This study showed that the expression of MS4A2 in the peripheral blood of patients with preeclampsia during early pregnancy was significantly lower than that of normal pregnant women, which also confirmed the role of mast cells in the pathogenesis of preeclampsia. Figure 9 summarizes the possible mechanisms of mast cell reduction leading to the pathogenesis of preeclampsia. During normal pregnancy, the interaction between uMCs, uNKs, trophoblasts, and regulatory T cells promotes pregnancy synergistically. The activation of uMCs and uNKs can release many mediators, including histamine, tryptase, and chymotrypsin. These mediators are directly or indirectly involved in the process of uterine spiral artery remodeling. The deficiency of UMCs can have a negative impact on pregnancy.

Researchers have made considerable efforts to identify biomarkers to predict the occurrence of preeclampsia early. The current strategy for predicting preeclampsia is based on a combination of baseline maternal factors, biophysical parameters, and placental-associated proteins (Poon and Nicolaides, 2014). The combination of these markers can identify preeclampsia, but unfortunately, they do not perform well in detecting all cases of preeclampsia (Pedrosa and Matias, 2011). Considering the existing evidence that the underlying immunopathology of preeclampsia may be derived from abnormal angiogenesis and transplantation, studying the immune response in the first three months may help develop a biomarker for predicting preeclampsia earlier. Furthermore, early recognition of preeclampsia in high-risk pregnant women may open a door to primary preventive strategies. As a result, we analyzed the peripheral blood immune effectors of normal and preeclamptic women to see if they might be used to predict preeclampsia in early pregnancy. This finding supports the idea that the pro-inflammatory immune response occurs before the actual diagnosis of preeclampsia, and that these indicators can be used in clinical practice to triage and manage high-risk populations in early pregnancy.

## Conclusion

In our study, we first identified 30 hub genes in the training cohort (GSE48424) by using WGCNA and DEGs and verified the expression of these genes in the validation cohort (GSE149437). Based on the above results and combined with bioinformatics technology, we identified three key genes (HDC, MS4A2, and SLC18A2). Then we established the prediction model of peripheral blood markers of preeclampsia and drew a nomogram to visualize the model. The calibration curve and ROC curve are used to verify the good predictive value of the above model. Finally, in the test cohort of clinical peripheral blood samples, the differential expression of three genes in preeclampsia peripheral blood was verified again, and MS4A2 was confirmed to be the only gene marker with a statistical difference between early-onset and late-onset preeclampsia. This study provides new biomarkers and a prediction model for preeclampsia.

## Strengths and Limitations

Peripheral biomarkers for preeclampsia are few and far between and most studies have focused on the placenta and decidua tissues of preeclampsia. There is still a lack of information on key genes in peripheral blood as potential biomarkers of preeclampsia. To our best knowledge, few studies have included WGCNA in similar cohorts. Another advantage of this study is that it is the first time to use a co-expression network to analyze related genes in the peripheral blood of patients with preeclampsia. WGCNA is more comprehensive and qualified to construct meaningful networks whether weighted or non-weighted. As a result, the analysis results are reliable and meaningful. The genes screened by WGCNA are not only related to the onset of preeclampsia, but also related to the severity of the disease. More importantly, peripheral blood samples are easy to obtain, repeatable, and low harm to pregnant women. Our preeclampsia prediction model based on differentially expressed genes has proved to be of good predictive value and is more valuable than using individual genes. At the same time, we visualized the prediction model for clinical use. In this study, we also identified MS4A2 in peripheral blood as a biomarker highly correlated with the onset and severity of preeclampsia.

The limitations of our research also require attention. The construction of related networks is only a preliminary analysis. Phenotype-specific networks such as transcription factor-gene networks have not been identified through in-depth research. Therefore, further large-sample studies and functional studies of key genes in preeclampsia are needed. Due to the small sample size used in this study, the results of this study might be limited when extrapolated. In addition, our study only verified the relationship between the levels of the three genes in peripheral blood and preeclampsia. We did not dynamically follow up on the changes of these genes throughout pregnancy. In the future study, we hope to dynamically follow up on the changes of the genes in peripheral blood of preeclampsia pregnant women and normal pregnant women throughout pregnancy. The functional effects will be further verified *in vitro* and in animal studies to clarify its mechanism in the pathogenesis of preeclampsia.

## Data Availability

The original contributions presented in the study are included in the article/Supplementary Material, further inquiries can be directed to the corresponding author.
